# Targeting lymph node delivery with nanovaccines for cancer immunotherapy: recent advances and future directions

**DOI:** 10.1186/s12951-023-01977-1

**Published:** 2023-07-07

**Authors:** Yueyi Li, Shen Li, Zedong Jiang, Keqin Tan, Yuanling Meng, Dingyi Zhang, Xuelei Ma

**Affiliations:** 1 Department of Biotherapy, Cancer Center, State Key Laboratory of Biotherapy, West China Hospital, Sichuan University, No.37, Guoxue Alley, Chengdu, 610041 China; 2 West China School of Stomatology, Sichuan University, Chengdu, Sichuan China; 3 West China School of Medicine, Sichuan University, Chengdu, China

**Keywords:** Nanovaccines, Lymph node, Cancer, Immunotherapy, Delivery

## Abstract

Although cancer immunotherapy is a compelling approach against cancer, its effectiveness is hindered by the challenge of generating a robust and durable immune response against metastatic cancer cells. Nanovaccines, specifically engineered to transport cancer antigens and immune-stimulating agents to the lymph nodes, hold promise in overcoming these limitations and eliciting a potent and sustained immune response against metastatic cancer cells. This manuscript provides an in-depth exploration of the lymphatic system’s background, emphasizing its role in immune surveillance and tumor metastasis. Furthermore, it delves into the design principles of nanovaccines and their unique capability to target lymph node metastasis. The primary objective of this review is to provide a comprehensive overview of the current advancements in nanovaccine design for targeting lymph node metastasis, while also discussing their potential to enhance cancer immunotherapy. By summarizing the state-of-the-art in nanovaccine development, this review aims to shed light on the promising prospects of harnessing nanotechnology to potentiate cancer immunotherapy and ultimately improve patient outcomes.

## Introduction

Cancer is a complex and heterogeneous disease that has been ceaselessly bringing up impenetrable challenges to the public health of human kinds. Though for decades we see countless advances in the field of cancer treatment, such as surgery, radiation therapy and chemotherapy, cancer remains a leading cause of death worldwide. The role of lymphatic system is indispensable for immune surveillance and tumor metastasis [1]. By entering the lymphatic vessels and transferring to the local lymph nodes, cancer cells can establish secondary tumors so that it can evade immune surveillance [1]. As the efficacy of cancer immunotherapy determined by the capability of immune cells in identifying and purging cancer cells, this requires the presence of cancer antigens to T cells by antigen-presenting cells (APCs) in the lymph nodes [2]. However, cancer antigens are often poorly immunogenic and are not efficiently delivered to the lymph nodes [2]. Nanovaccines, designed to deliver cancer antigens and immune-stimulating agents to the lymph nodes, have the potential to overcome these limitations and induce a potent and sustained immune response against metastatic cancer cells.

Tumor immunotherapy has gradually become an ever-concrete strategy against cancer recent years, with several immunotherapeutic agents receiving approval from regulatory agencies for the treatment of various cancers. These agents include immune checkpoint inhibitors, chimeric antigen receptor (CAR) T-cell therapy, and tumor-infiltrating lymphocytes (TILs) therapy. Immune checkpoint inhibitors work by blocking proteins on the surface of immune cells, such as cytotoxic T-lymphocyte-associated protein 4 (CTLA-4) or programmed cell death protein 1 (PD-1), which can restrict the immune system from identifying and purging and attacking cancer cells [3]. FDA-approved immune checkpoint inhibitors in cancer immune oncology have been showed in Table 1. By blocking these proteins, immune checkpoint inhibitors boost the immune system for better ability in identifying the purging the cancer cells. CAR-T works by genetically reengineering one’s own T-cells so that they can express a receptor which recognizes and attacks cancer cells. These re-engineered T-cells are then injected back into the patient, so that they can target and destroy cancer cells [4]. TILs therapy involves isolating immune cells from a patient’s tumor and expanding them in the laboratory before injecting them back into the patient. These expanded TILs can then target and destroy cancer cells [5].

**Table 1:** FDA-approved immune checkpoint inhibitors in cancer immune oncology

Inhibitor	Target	Ligand/receptor	Year of approval	Cancer types
Atezolizumab (Tecentriq)	PD-L1	PD-1	May-2016	Bladder cancer/ NSCLC
Avelumab (Bavencio)	PD-L1	PD-1	November-2015	Bladder cancer/ Merkel cell carcinoma
Durvalumab (Imfinzi)	PD-L1	PD-1	February-2016	Bladder cancer
Nivolumab (Opdivo)	PD-1	PD-L1, PD-L2	March-2015	Bladder cancer/ Head and neck cancer (squamous cell carcinoma)/ Classical Hodgkin lymphoma/ Melanoma/ Mismatch repair deficient and microsatellite instability-high colorectal cancer/ NSCLC / Renal cell (kidney) cancer
Pembrolizumab (Keytruda)	PD-1	PD-L1, PD-L2	September2014	Bladder cancer/ Head and neck cancer (squamous cell carcinoma)/ Classical Hodgkin lymphoma/ Melanoma/ Mismatch repair deficient and microsatellite instability-high solid tumors / NSCLC
Cemiplimab (Libtayol)	PD-1	PD-L1, PD-L2	September-2018	NSCLC/ Squamous Cell Carcinoma of skin/ Basal Cell Carcinoma
Ipilimumab (Yervoy)	CTLA-4	Cd80/CD86	March-2011	Melanoma

NSCLC: Non-small cell lung cancer.

While these immunotherapeutic agents have made incredible progress in tackling different types of cancer, among which includes melanoma, non-small cell lung cancer, and bladder cancer, they are associated with considerably significant toxicities and limitations yet follow [6]. For instance, immune checkpoint inhibitors can cause autoimmune reactions, so that the immune system attacks normal tissues, leading to side effects such as rash, diarrhea, and pneumonitis [7]. CAR T-cell therapy could also bring up severe side effects, including cytokine release syndrome (CRS) and neurotoxicity [4].

In addition to these toxicities, there are also limitations related to the ability of these therapies to target solid tumors. While immunotherapy has made incredible progress success in treating some types of cancer, such as melanoma, efficacy diminishes greatly, when it is used against other types of cancer, such as pancreatic cancer and glioblastoma [8, 9]. This may be due, in part, to the difficulty in accessing and targeting solid tumors, which can be protected by a variety of physical and immunological barriers. Despite these limitations, tumor immunotherapy continues to show promise as a potential strategy for improving cancer treatment outcomes. Researchers are continuing to explore novel approaches for enhancing the efficacy of immunotherapy, including combination therapies, novel immunotherapeutic agents, and targeted delivery systems.

Tumor vaccines represent a potential strategy for stimulating the immune system to identifying and purging cancer cells. Tumor vaccines work by presenting tumor-specific antigens to the immune system, where the immediate immune response against cancer cells would be triggered simultaneously [10]. The classification of the tumor vaccines is based on the type of antigen used, which can be either whole-cell or specific antigen-based [11]. Whole-cell tumor vaccines use the patient’s own cancer cells as the source of antigen, while specific antigen-based vaccines use specific proteins or peptides that are expressed by cancer cells [12]. Despite the potential benefits of tumor vaccines, these therapies face several limitations. One of the major limitations of tumor vaccines is the difficulty in identifying tumor-specific antigens that can be used to stimulate an immune response [13]. Another limitation is the potential for immune tolerance, in which the immune system becomes tolerant to tumor-specific antigens, leading to a lack of response to therapy [10, 14]. Additionally, tumor vaccines can be limited by the heterogeneity of tumors, which can make it difficult to identify a single antigen expressed by all cancer cells [13, 15].

One potential strategy for addressing the limitations of tumor vaccines is the targeted delivery of antigens to lymph nodes. Lymph nodes play a critical role in the immune response by serving as a site of antigen presentation and immune cell activation. Therefore, delivering tumor-specific antigens directly to lymph nodes can potentially enhance the immune response against cancer cells [16]. Targeted lymph node delivery can be achieved through the use of carrier molecules, such as liposomes or nanoparticles, that are designed to selectively target lymph nodes [16]. These carrier molecules can be modified with targeting ligands or antibodies to increase their specificity for lymph nodes.

**Fig. 1:**
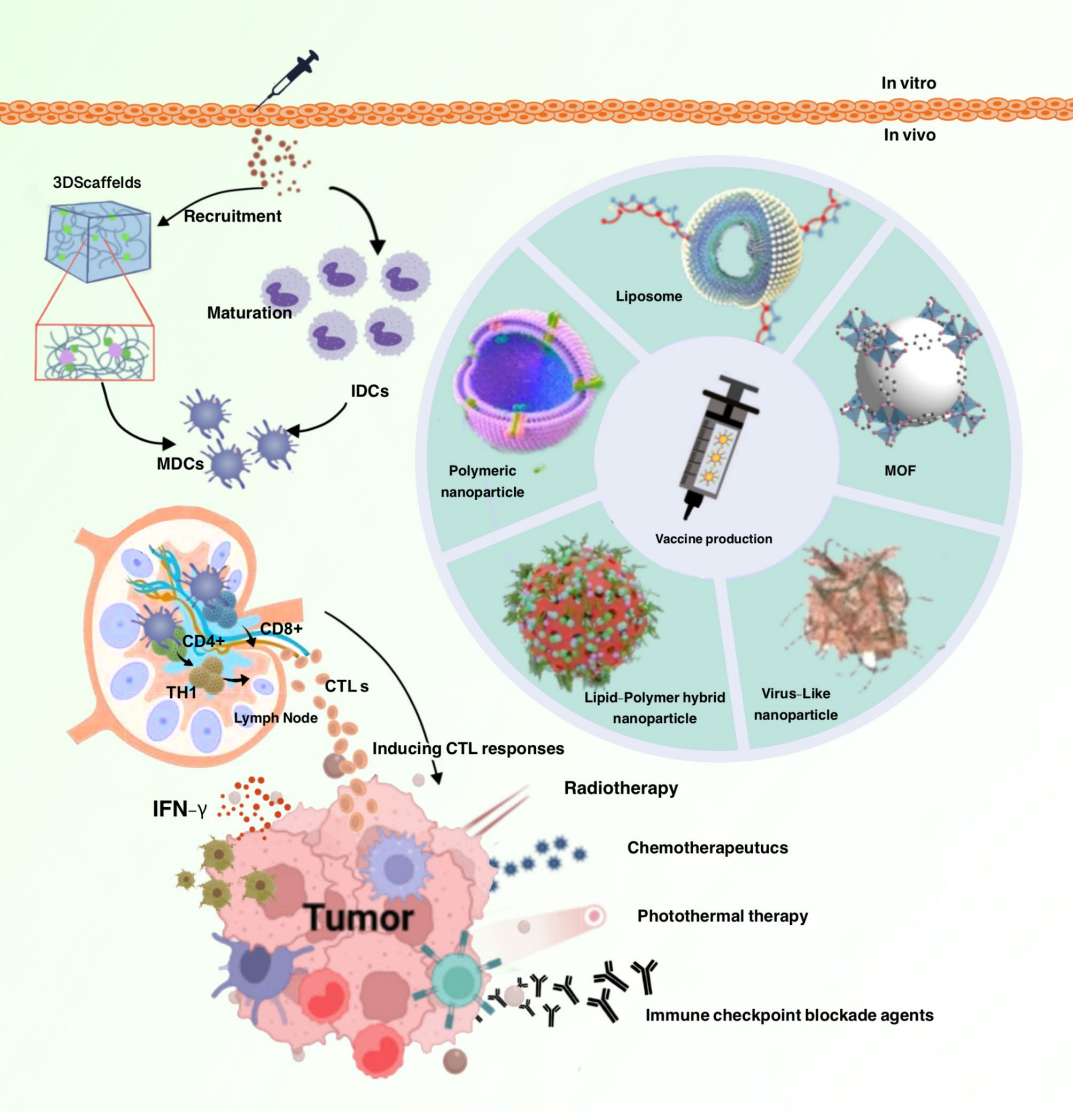
Nanovaccines in cancer immunotherapy

Tumor immunotherapy represents a promising strategy for improving cancer treatment outcomes. Tumor vaccines offer a potential approach for stimulating the immune system to recognize and attack cancer cells, but face several limitations related to antigen identification, immune tolerance, and tumor heterogeneity [13]. Targeted lymph node delivery of tumor-specific antigens stands as a potential strategy for enhancing the efficacy of tumor vaccines [16]. This article provides a comprehensive review of recent advances and future directions in targeted lymph node delivery systems for cancer treatment (Fig. 1).

## Lymphatic system in tumor immune system

### DC cells

Dendritic cells (DCs) play a crucial role in both the innate and adaptive immune system responses. Specifically, they are capable of presenting tumor antigens and expressing highly stimulating molecules for effectively activating cytotoxic T lymphocytes (CTLs) so that cancer immunotherapy could be conducted [17]. In order to incur a powerful CTL response, immature DCs (iDCs) must first take up, process, and deliver antigens to major histocompatibility complex (MHC) molecules located on their surface. This antigen-MHC complex then activates naïve T cells which mature into CTLs [18]. To facilitate antigen binding, iDCs undergo a phenotypic and functional transformation and differentiate into mature DCs (mDCs). Such transformation is conducted along with the boost and regulation from a variety of receptors such as chemokine receptors, adhesion receptors, and costimulatory molecules [19]. Additionally, with the high expression of MHC molecules on mDCs, the activation of antitumor immune responses is hereby optimized [20].

**Fig. 2:**
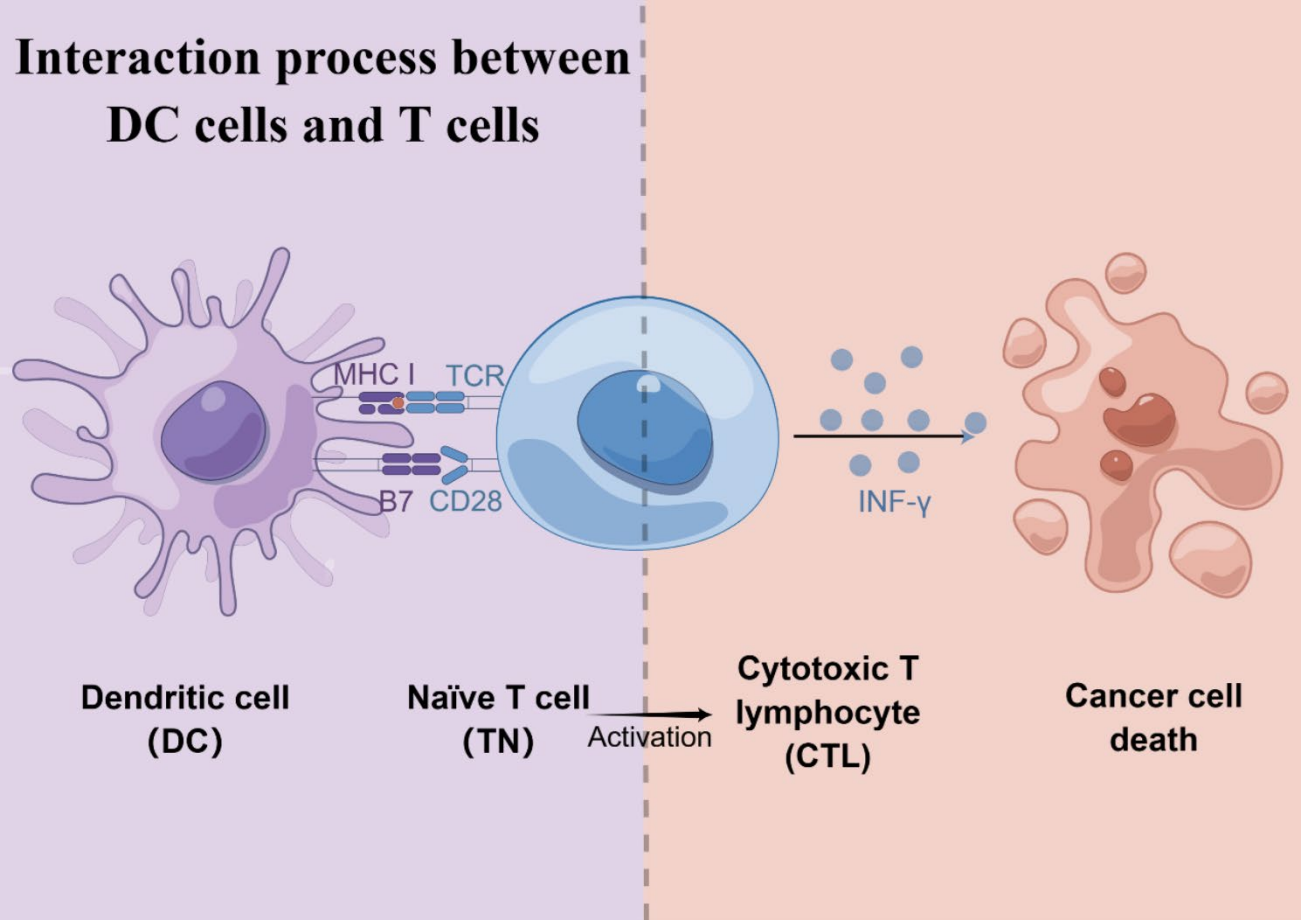
Interaction between DC cells and T cells during antigen presentation. (By Figdraw)

Antigen presentation involves internalization, degradation, and loading of antigenic peptides onto MHC molecule of APCs [21]. Among all APCs, DCs are considered the most significant, capable of delivering exogenous antigens [22]. DCs is important in the development of adaptive immune responses to suppress tumors, as it can collect and render antigenic peptides onto MHC-I molecules through the cross-presenting pathway [23]. T-cell receptors (TCRs) recognize antigenic peptides bound to MHC-I on DC surfaces, priming and activating CD8 + T cells, which are well-known for producing interferon-𝛾 (IFN-𝛾) for antitumor immunity [24]. The ultimate effector T cells are activated by DCs [25]. Thus, the T-cell activation process is influenced by the intensity and duration of interactions between DCs and T cells. Additionally, building effective intercellular communication between DCs and T cells is dependent on the upregulation of costimulatory molecules on DC surfaces [26] (Fig. 2).

### T cells

In the MHC-II pathway, exogenous antigens are internalized into endosomes, where they undergo proteolytic cleavage by enzymes. The resulting peptides are then presented to MHC-II molecules within the endosome and subsequently delivered to the cell membrane, where they react with CD4 + T cells for the initialization of the immune response. In contrast, in the classical MHC-I pathway, endogenous antigens are degraded by proteasomes in the cytosol and delivered into the endoplasmic reticulum. With the assistance of the transporter associated with antigen processing (TAP), the processed antigenic peptides are loaded onto MHC-I molecules. The MHC-I-peptide complexes are then delivered to the cell membrane, and through engagement with CD8 + T cells it achieves immune activation.

The molecular mechanisms governing cross-presentation, a process that allows for the presentation of exogenous antigens on MHC-I molecules, are not as well understood as the pathways involved in MHC-II and classical MHC-I presentation and remain the subject of debate. Broadly, there are two main pathways implicated in cross-presentation: the cytosolic pathway and the vacuolar pathway [27]. In the cytosolic pathway, exogenous antigens are internalized into endosomes and broken down by proteases. The resulting antigen peptides can dodge the endosomes and enter the cytoplasm, through either disruption of the endosomal membrane or via transportation mediated by ER-associated degradation [28]. However, the precise mechanisms and extent to which these processes contribute to antigen transport into the cytosol are not fully understood. Once in the cytoplasm, the escaped peptides are further processed by the proteasome and delivered to the ER with the assistance of TAP, where they are loaded onto MHC-I molecules. Subsequently, the MHC-I-peptide complexes are transported to the cell membrane, where they engage with CD8 + T cells to initiate an immune response. Alternatively, peptides digested by the proteasome can be transported back to the endosomes and integrated into MHC-I molecules within the endosomal compartments, which are then transported to the cell membrane for CD8 + T cell activation. In the vacuolar pathway, in contrast, antigens are degraded within endosomes and directly loaded onto MHC-I molecules [29]. The resulting MHC-I-peptide complexes are transported to the cell membrane to stimulate CD8 + T cell responses. In this pathway, it is generally believed that the MHC-I molecules in endosomal compartments would be recycled from cell surface complexes.

### Lymph node

**Fig. 3:**
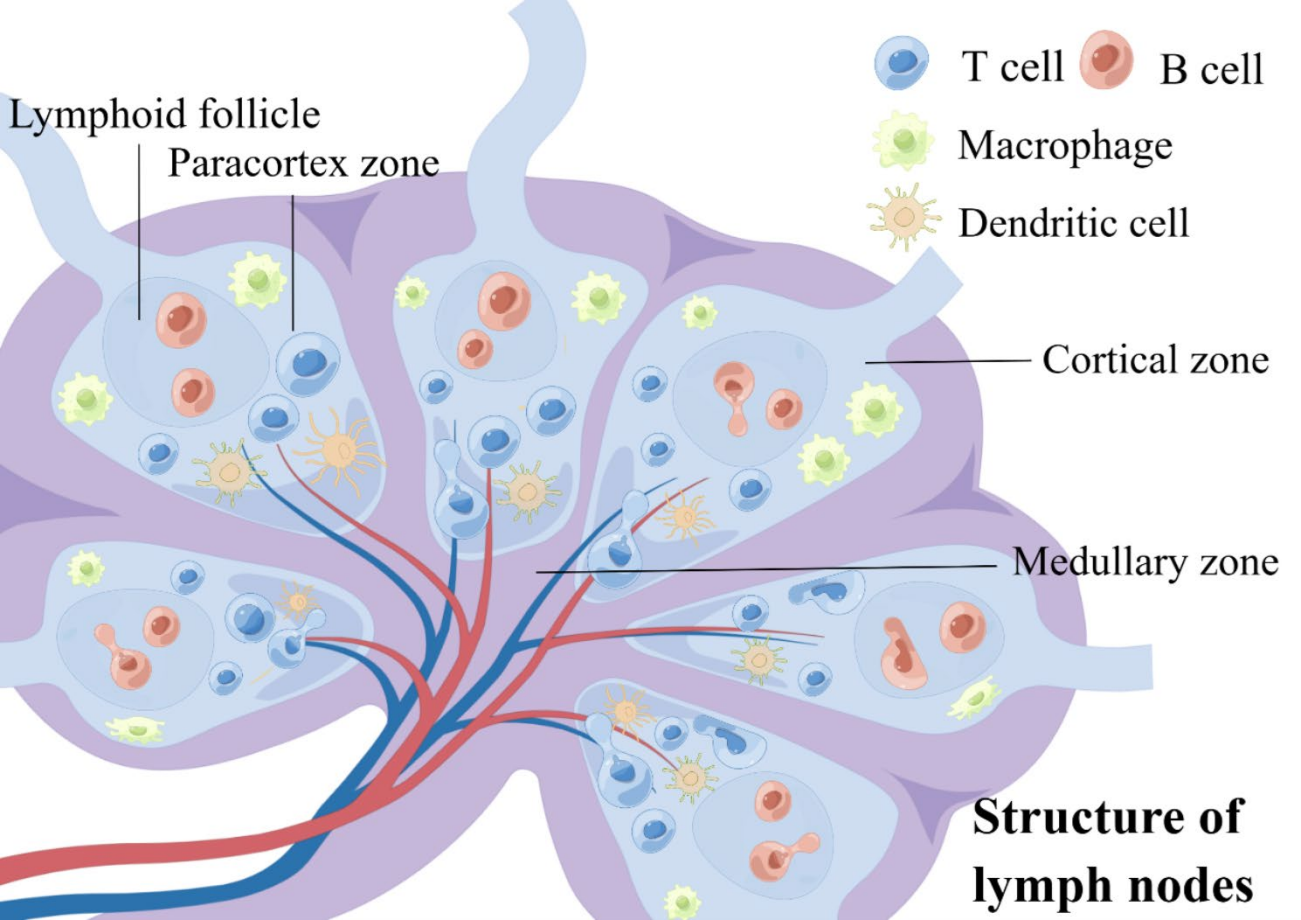
The structure of lymph nodes. (By Figdraw)

Lymph nodes are small, oval-shaped bodies of lymphatic tissue of varying sizes, interspersed with and connected to lymphatic vessels, through which thousands of lymph nodes are connected throughout the body [30]. The cortex of the lymph nodes consists of a superficial cortex containing B cells and a paracortex zone rich in T cells. Inside the lymph nodes is the medulla, which contains the medullary cords and medullary lymphatic sinuses. The medulla contains mainly B cells, plasma cells and macrophages [31]. The lymph nodes receive lymphatic fluid from cells and tissues, and through the phagocytosis of phagocytes in the lymphatic sinuses and the action of immune molecules such as antibodies, they can kill pathogenic microorganisms and remove foreign bodies, thus serving to purify the lymphatic fluid and prevent the spread of pathogens [32]. Under normal conditions, most lymphocytes are recirculating lymphocytes and only a few divided and proliferate in the lymph nodes. Lymphocytes in the blood are recruited to the lymph nodes mainly through the high endothelial venules and lymphatic vessels, into the paracortex of the lymph nodes and then converge via the lymphatic sinuses into the efferent lymphatic vessels. The numerous lymph nodes throughout the body, particularly the thymus, bone marrow, tonsils and spleen, are important supplementary sources of lymphocytes. The lymph nodes are rich in various types of immune cells, which can facilitate the capture of antigens, the transmission of antigenic information and cell activation and proliferation [33]. B cells are stimulated by external antigens or antigens presented by the APC to differentiate and proliferate, generating large numbers of plasma cells to form germinal centers. T cells can also differentiate and proliferate in the lymph nodes as sensitized lymphocytes (Fig. 3). Follicular dendritic cells have abundant Fc receptors on their surface, which can trap antigen-antibody complexes and retain antigens in the follicle for long periods of time, helping to form and maintain B cells and memory cells, and aiding secondary immunity [34]. The aggregation and maturation of immune cells help lymph nodes perform their function of filtering lymph fluid and immune response.

The lymphatic system is an important pathway for cancer cells to escape from the primary lesion [35]. The earliest stage of lymphatic metastasis is the sentinel lymph node (SLN), defined as the first lymph node that metastasizes directly from the primary tumor. SLNs have altered biological features compared to normal lymph nodes, including both an increase in lymphatic vessels and increased lymphatic flow, structural remodeling of small high endothelial veins, and a decrease in effector lymphocytes [33, 36, 37]. These alterations contribute to the formation of a tumor microenvironment for more cancer cells to enter and survive [38]. Lymph node metastasis (LNM) has been shown to be an indispensable prognostic feature in clinical staging and treatment decisions for cancer [39]. Lymph node dissection has become a necessary surgery for several high mortality tumors such as gastric cancer, breast cancer and melanoma [40–42].

Due to the linkage of lymphatic fluid and high endothelial venules, LNs are a dedicated location for a rapidly reacting immune response. Through the transport of activated APCs and soluble antigens from peripheral tissues, LNs promote the activation and proliferation of antigen-specific T and B cells and induce the maturation of DCs [43]. Due to this feature of lymphatic capillaries draining interstitial fluid from local tissues, we can design specific LN targeting strategies to indirectly target LNs using drug delivery systems and enhance the immune effects of LNs as a new approach to cancer immunotherapy.

## Nanovaccines for lymph nodes

### The routes for the vaccine to go towards the lymph nodes

It is crucial to systematically analyze different administration routes for vaccines in which lymph node-targeted vaccines achieve optimal immune activation. Some vaccines are administered through the oral or interstitial injection routes, including subcutaneous, intramuscular, and intradermal injections [44]. Though the properties of low cost and improved patient adherence of oral vaccines earn popularity in developing countries. Lymph node targeting can hardly achieve its optimal effectiveness due to the harsh gastrointestinal environment of the human body and self-downregulation of the immune response [45]. In recent years, such drawbacks incur more and more focus on the possibility of direct intranodal injection of vaccines for LN targeting, particularly for DC-based vaccines [46]. However, this method faces technical challenges in locating lymph nodes, among which is that the delicate structure of LNs and cytokine gradients inside may be irreparably damaged by direct injection [45]. Hence, traditional interstitial vaccination remains the primary method of vaccine administration.

Compared with intranodal vaccination, interstitial vaccination involves a longer pathway in circulation through the lymphatic vessels, which can negatively impact the efficacy of vaccines. To better understand how vaccines are delivered to the lymph nodes, two possible pathways have been identified. During subcutaneous, intradermal, or intramuscular vaccination, macrophages and DCs, acting as antigen-presenting cells, capture antigens within peripheral tissues. These APCs then digest and present the antigens as MHC molecules. The activated APCs move from the interstitial space and cross lymphatic endothelial cells into the lymphatic vessels with interstitial fluid. For instance, due to the upregulation of CCR7 signaling, mDCs undergo migration towards lymphatic vessels [47].

For the activation of the adaptive immune response, APCs travel from the site of vaccination to the draining lymph nodes via lymphatic vessels, with the help extended by chemokine gradients and luminal valves [48]. APCs in the lymph nodes encounter and present antigens to naïve lymphocytes, initiating the corresponding adaptive immunity. However, those antigens unprocessed by APCs in the periphery can rely only on the passive drain into the lymphatic vessels, through which they are unidirectionally delivered to lymph nodes. With the absence of the digestion and presentation by APCs, lymph node’s capability in targeting is generally weaker [49]. Furthermore, the effectiveness of vaccine delivery to the lymph nodes can be diminished by the clearance of vaccines through blood capillaries and potential uptake of vaccines by different cellular entities via endocytosis [50]. To overcome these challenges, efforts have been made to enhance the efficiency of vaccine delivery through the design of specialized carriers.

The nanovaccines can reach lymph nodes in two ways, either by moving through the interstitium via diffusion and convection, or by being transported by APCs like DCs or macrophages. In the lymph nodes, DCs deliver the antigen to T cells so that further proliferation and differentiation can be finalized, ultimately leading to the activation of lymphocytes that provide immunity in the body [51, 52]. Nanomaterials are a promising way to improve the targeting efficiency of the lymph nodes and enhance the antitumor immunity effect. As an example, a polymer-based nanovaccines incorporating a toll-like receptor agonist adjuvant demonstrated prolonged retention in the draining lymph nodes, with a duration of up to 20 days and showed a better lymph nodes’ targeting and accumulation effect than small molecules alone [53]. Superior targeting activity and antitumor immune efficacy were observed when utilizing nanovaccines complexes loaded with adjuvants and neoantigens encapsulated within synthetic high-density lipoprotein nanodiscs [54].

### Antigen transport in lymph nodes

Upon entry into the lymph nodes via afferent lymphatic vessels, the presence of antigens or APCs derived from vaccine administration or antigen injection sites stimulates a robust immune response, leading to the aggregation of lymphocytes within lymphoid lobules. When activated by antigens, lymphocytes differentiate into effector cells or memory cells and depart from the lymph nodes through lymphatic vessels. This departure is facilitated by the expression of distinct chemokine receptors, initiating adaptive immune responses. These responses offer targeted and enduring protection by eliminating relevant pathogens and antigens [55].

Lymphatic fluid flows through lymphatic vessels and passes through the subcapsular sinuses and cortical sinus before migrating to different areas within the lymph node [56]. Antigen-carrying APCs, such as DCs, face barriers in the form of lymphatic endothelial cells between the T cell inhabited cortex zone and subcapsular sinuses, which they cannot cross without the aid of chemokine gradients along the sinus floor [57]. On the other hand, resident immature antigen-presenting cells within the lymph nodes play a crucial role in the transportation of free vaccine antigens originating from peripheral tissues [58]. The transportation time of antigens significantly depends on their size, with small antigens transiting easily from the subcapsular sinus and large antigens facing challenges as conduit system functions to exclude their entries [59–61]. Macrophages inside the subcapsular sinus take responsibility for capturing and digesting trapped large antigens to assist their transportation to B cells in the underlying follicles [48]. Upon maturation and activation within the lymph nodes, lymphocytes and antibodies concentrate within the inner medulla before exiting through the efferent lymphatic vessels. This mechanism ensures targeted and durable defense against relevant pathogens and antigens.

Tumor-draining lymph nodes are important for presenting tumor antigens to the immune system, and are a target of importance for cancer vaccines. However, the structure and composition of these lymph nodes can be altered by the presence of cancer, which can suppress resident dendritic cells and promote lymphangiogenesis [62]. Tumor-derived substances can also disrupt the presentation of tumor antigens and activate regulatory T cells [63, 64]. The overexpression of chemokine receptors can stimulate the movement of malignant cells via the lymphatic capillaries [65–67]. Through the specific targeting of lymph nodes associated with tumors, it is possible to modulate the immunology response to promote anti-tumor immunity and prevent immune tolerance, ultimately enhancing the effectiveness of cancer vaccines.

### Nanovaccines delivery systems

Nanovaccines consist of antigens, adjuvant, and/or nanocarriers [68]. Nanocarriers serve multiple functions, such as shielding the antigens and/or adjuvant from enzymatic degradation, controlling the release of cargo, and augmenting immune responses compared to free antigens and adjuvant [69]. Nanocarriers are generally particles with a size ranging from 10 to 100 nm and sometimes up to 1000 nm [70]. polymer-based nanomedicines and liposomes can be tailored to deliver antigens or viral peptides effectively to antigen-presenting cells and trigger memory T-cell responses against tumors [71]. As the safety and efficacy of nanovaccines are gradually being discovered in animal studies, targeting lymph nodes characterized by this approach can lead to long-lasting and specific immunity. Thus, phase I, II and III clinical trials for the population have been initiated with the aim of bringing this promising new tumor immunity approach to the clinic. We concluded the clinical trial of the nanovaccines in Table 2.

**Table 2:** Clinical trial of the nano vaccine

Catalog	Name	Status	Identifier	Phase	Sponsor	Disease	Cancer type
Liposome vaccine	Tecemotide (L-BLP25)	Completed	NCT00828009	Phase 2	ECOG-ACRIN Cancer Research Group	Non-Small Cell Lung Cancer	Stage IIIA Stage IIIB
		Completed	NCT00960115	Phase 1/ Phase 2	Merck KGaA, Darmstadt, Germany	Non-small Cell Lung Cancer	Stage III
		Completed	NCT00409188	Phase 3	EMD Serono	Non-small Cell Lung Cancer	Stage III
		Terminated	NCT01015443	Phase 3	Merck KGaA, Darmstadt, Germany	Non-small Cell Lung Cancer	Stage III
		Terminated	NCT00925548	Phase 3	EMD Serono	Breast Cancer	-
		Completed	NCT00157209	Phase 2b	Merck KGaA, Darmstadt, Germany	Non-Small-Cell Lung	-
		Completed	NCT00157196	Phase 2	Merck KGaA, Darmstadt, Germany	Non-Small Cell Lung Cancer	Stage III
		Completed	NCT01496131	Phase 2	EMD Serono	Prostate Cancer	Untreated, Intermediate and High Risk
		Completed	NCT01094548	Phase 2	Merck KGaA, Darmstadt, Germany	Multiple Myeloma	no Symptoms /No Chemotherapy
		Terminated	NCT01423760	Not Applicable	Merck KGaA, Darmstadt, Germany	Non-Small Cell Lung Cancer / Multiple Myeloma	-
	RNA-lipid Particle (RNA-LP) Vaccines	Recruiting	NCT04573140	Phase 1	University of Florida	Glioblastoma	-
	W_ova1 Vaccine	Active, not recruiting	NCT04163094	Phase 1	University Medical Center Groningen	Ovarian Cancer	-
	NY-ESO-1 peptide vaccine	Completed	NCT01673217	Phase 1	Roswell Park Cancer Institute	Recurrent Fallopian Tube Cancer/ Recurrent Ovarian /Epithelial Cancer Recurrent Primary Peritoneal Cavity Cancer	-
	DPX-0907	Completed	NCT01095848	Phase 1	ImmunoVaccine Technologies, Inc. (IMV Inc.)	Ovarian Neoplasms /Breast Neoplasms/ Prostatic Neoplasms	HLA-A2 Positive Advanced Stage
	DOTAP liposome vaccine	Not yet recruiting	NCT05264974	Phase 1	University of Florida	Melanoma	-
	ONT-10	Completed	NCT01556789	Phase 1	Cascadian Therapeutics Inc.	Solid Tumors	Stage III or IV
		Completed	NCT01978964	Phase 1b	Cascadian Therapeutics Inc.	Solid Tumors	Stage III or IV
	PDS0101	Recruiting	NCT04580771	Phase 2	M.D. Anderson Cancer Center	Locally Advanced Cervical Squamous Cell Carcinoma, Not Otherwise Specified	Stage IB3-IVA
		Recruiting	NCT05232851	Phase 1 /Phase 2	Mayo Clinic	Locally Advanced Human Papillomavirus-Associated Oropharynx Cancer	-
	autologous tumor cell vaccine	Completed	NCT00020462	Phase 1	National Cancer Institute (NCI)	Lymphoma	-
	Lipovaxin-MM	Completed	NCT01052142	Phase 1	Lipotek Pty Ltd	Melanoma	-
	Oncoquest-CLL vaccine	Active, not recruiting	NCT01976520	Phase 1	XEME Biopharma Inc.	Chronic Lymphocytic Leukemia (CLL)	-
	polyvalent melanoma vaccine	Completed	NCT00004104	Phase 2	NYU Langone Health	Melanoma (Skin)	Stage III
	Liposomal Doxorubicin	Completed	NCT00923936	Phase 2	National Cancer Institute (NCI)	Sarcoma, Kaposi	-
Polymeric nanoparticles	Cetuximab nanoparticles	Unknown	NCT03774680	Phase 1	Ahmed A. H. Abdellatif	Colon Cancer/ Colo-rectal Cancer	-
	Docetaxel-PNP	Completed	NCT01103791	Phase 1	Samyang Biopharmaceuticals Corporation	Advanced Solid Malignancies	-
	Quercetin-encapsulated PLGA-PEG nanoparticles (Nano-QUT)	Not yet recruiting	NCT05456022	Phase 2	Cairo University	Oral Cancer	-
	Docetaxel-PNP	Completed	NCT02274610	Phase 1	Samyang Biopharmaceuticals Corporation	Solid Tumor	-
	CRLX101	Terminated	NCT03531827	Phase 2	National Cancer Institute (NCI)	Metastatic Castration Resistant Prostate Cancer Prostate Neoplasms	-
Virus-like particles	HPV-16/18 L1 virus-like particle/AS04 vaccine	Completed	NCT00128661	Phase 3	GlaxoSmithKline	Prevent Cervical Cancer/ Precancerous Condition	-
	HPV-16/18 vaccine	Completed	NCT02740777	Phase 2	Shanghai Zerun Biotechnology Co.,Ltd	Human Papillomavirus Cervical Intraepithelial Neoplasia Persistent Infection	-
		Completed	NCT02733068	Phase 3	Shanghai Zerun Biotechnology Co.,Ltd	Cervical Intraepithelial Neoplasia	-
	9vHPV Vaccine	Completed	NCT01651949	Phase 3	Merck Sharp & Dohme LLC	Genital Warts /Anal Cancer/ Anal Intraepithelial Neoplasia	-
		Active, not recruiting	NCT04199689	Phase 3	Merck Sharp & Dohme LLC	Papillomavirus Infections	-
		Completed	NCT03546842	Phase 3	Merck Sharp & Dohme LLC	Papillomavirus Infections Uterine Cervical Neoplasms Vulvar Neoplasms Vaginal Neoplasms	-
		Active, not recruiting	NCT05285826	Phase 3	Merck Sharp & Dohme LLC	Papillomavirus Infections	-
	Quadrivalent HPV for types 6, 11, 16 and 18	Completed	NCT01101750	Phase 4	Medstar Health Research Institute	Cervical Cancer/ Hpv /Warts	-
		Completed	NCT00635830	Phase 1	Merck Sharp & Dohme LLC	HPV Infections	-
		Completed	NCT00337428	Phase 3	Merck Sharp & Dohme LLC	Neoplasms, Glandular and Epithelial Diphtheria Tetanus Whooping Cough Poliomyelitis	-
		Completed	NCT00834106	Phase 3	Merck Sharp & Dohme LLC	HPV Infections	-
		Active, not recruiting	NCT03728881	Phase 3	National Cancer Institute (NCI)	Human Papillomavirus-Related Cervical Carcinoma	-
		Completed	NCT00380367	Phase 3	Merck Sharp & Dohme LLC	Papillomavirus Infections	-
		Completed	NCT04711265	-	Kenya Medical Research Institute	HPV Infection HIV-1-infection	-
		Completed	NCT00496626	Phase 3	Merck Sharp & Dohme LLC	Papillomavirus Infections	-
	Gardasil	Active, not recruiting	NCT00092534	Phase 3	Merck Sharp & Dohme LLC	Cervical Cancer Genital Warts	-
	Agonistic Anti-OX40 Monoclonal Antibody INCAGN01949	Terminated	NCT04387071	Phase 1/ Phase 2	University of Southern California	Pancreatic Cancer and Other Cancers Except Melanoma	Stage IV
	V501	Completed	NCT01544478	Phase 4	Merck Sharp & Dohme LLC	Cervical Cancer Cervical Intraepithelial Neoplasia Adenocarcinoma in Situ	-
		Completed	NCT00092495	Phase 3	Merck Sharp & Dohme LLC	Cervical Cancer Genital Warts	-
		Completed	NCT00092495	Phase 3	Merck Sharp & Dohme LLC	Cervical Cancer Genital Warts	-
		Completed	NCT00517309	Phase 3	Merck Sharp & Dohme LLC	Cervical Cancer Genital Warts	-
		Completed	NCT00092482	Phase 3	Merck Sharp & Dohme LLC	Cervical Cancer Genital Warts	-
		Completed	NCT00092547	Phase 3	Merck Sharp & Dohme LLC	Papillomavirus Infections	-
		Completed	NCT01862874	Phase 3	Merck Sharp & Dohme LLC	Anogenital Human Papilloma Virus Infection /Condyloma Acuminata	-
		Completed	NCT02576054	Phase 3	Merck Sharp & Dohme LLC	Anogenital Human Papilloma Virus Infection Condyloma Acuminata	-
		Completed	NCT00411749	Phase 2	Merck Sharp & Dohme LLC	HPV Infections	-
	V503	Completed	NCT00543543	Phase 3	Merck Sharp & Dohme LLC	Cervical Cancer Vulvar Cancer Vaginal Cancer Genital Warts Human Papillomavirus Infection	-
		Completed	NCT03158220	Phase 3	Merck Sharp & Dohme LLC	Cervical Cancer Vulvar Cancer Vaginal Cancer Genital Warts Human Papillomavirus Infection	-
		Active, not recruiting	NCT04635423	Phase 3	Merck Sharp & Dohme LLC	Neoplasms	-
		Completed	NCT01254643	Phase 3	Merck Sharp & Dohme LLC	Papillomavirus Infections	-
		Completed	NCT01984697	Phase 3	Merck Sharp & Dohme LLC	Human Papillomavirus Infection	-
		Active, not recruiting	NCT03903562	Phase 3	Merck Sharp & Dohme LLC	Papillomavirus Infections	-
		Completed	NCT02114385	Phase 3	Merck Sharp & Dohme LLC	Papilloma Viral Infection	-
		Completed	NCT01073293	Phase 3	Merck Sharp & Dohme LLC	Papillomavirus Infections	-
		Completed	NCT00988884	Phase 3	Merck Sharp & Dohme LLC	Human Papillomavirus Infection	-
		Completed	NCT00943722	Phase 3	Merck Sharp & Dohme LLC	Cervical Cancers/ Vulvar Cancer/ Vaginal Cancer/ Genital Lesions/ PAP Test Abnormalities/ HPV Infections	-
		Completed	NCT01047345	Phase 3	Merck Sharp & Dohme LLC	Cervical Cancers/ Vulvar Cancers/ Vaginal Cancers/ Genital Warts	-
		Active, not recruiting	NCT02653118	-	Merck Sharp & Dohme LLC	Cervical Cancer/ Vulvar Cancer/ Vaginal Cancer/ Genital Warts/ Human Papillomavirus Infection	-
		Active, not recruiting	NCT03998254	Phase 3	Merck Sharp & Dohme LLC	Papillomavirus Infections	-
	V504	Completed	NCT00551187	Phase 2	Merck Sharp & Dohme LLC	Cervical Cancer Vulvar Cancer Vaginal Cancer Genital Warts Human Papillomavirus Infection	-
	V505	Completed	NCT00520598	Phase 2	Merck Sharp & Dohme LLC	Cervical Cancer Vulvar Cancer Vaginal Cancer Genital Warts Human Papillomavirus Infection	-
	Recombinant Human Papillomavirus Bivalent	Recruiting	NCT05045755	-	Jun Zhang	Cervical Intraepithelial Neoplasia Cervical Cancer Vaginal Intraepithelial Neoplasia Vulvar Intraepithelial Neoplasia	-
	AAVLP-HPV	Completed	NCT03929172	Phase 1	2 A Pharma AB	Papillomavirus Infections	-
	Gardasil® HPV vaccine	Active, not recruiting	NCT04953130	Phase 4	London School of Hygiene and Tropical Medicine	HPV Infection Vaccine Preventable Disease	-
		Active, not recruiting	NCT00092534	Phase 3	Merck Sharp & Dohme LLC	Cervical Cancer Genital Warts	-
	Cervarix	Completed	NCT00586339	Phase 2	GlaxoSmithKline	Infections, Papillomavirus	-

#### Liposomes

Liposomes are a type of lipid-based nanovesicles that have been approved for clinical use in cancer treatment due to their ability to protect antibodies from degradation, high biocompatibility, and efficient distribution [72]. They are capable of delivering a wide range of cancer drugs, including polypeptide, nucleic acid, and antibody drugs, making them effective for delivering cancer immunotherapies that activate either humoral or cellular immune responses [73, 74]. Liposomes are versatile and can be used for various immunotherapeutic cancer treatments, such as checkpoint blockade and vaccination [75]. Researchers have developed a liposomal formulation of vemurafenib for treating subcutaneous melanoma by encapsulating it in modified liposomes containing the peptide TD. Vemurafenib has been demonstrated to selectively inhibit A375 melanoma cells in in vitro experiments. Furthermore, administration via liposomes through the skin was found to be more effective compared to oral or intravenous administration, as it did not cause damage to the liver, kidney, or lung. Furthermore, the incorporation of the TD peptide modification onto the liposomes significantly enhanced the transdermal delivery of vemurafenib [76]. There are diverse immune-related cells that liposomes target in the field of immune-oncology, leading to the activation of either cellular or humoral immune responses, such as NK cells, T cells, dendritic cells, fibroblasts etc.

#### Polymeric nanoparticles

Nowadays, More and more focus has been put into using polymer-based nanomedicines for diagnosing and treating various diseases [77]. Among these, polymer-based tumor nanovaccines have been of particular interest for cancer immunotherapy due to their structural flexibility and biodegradability [78, 79]. By modifying the polymers with targeting ligands or sensitive bonds that can be cleaved under specific conditions, such as intracellular acidic pH or glutathione (GSH), the immune efficacy of the nanovaccine can be enhanced [79, 80]. Nanocarriers offer the possibility to stabilize antigens or adjuvants within the body, facilitate precise drug delivery through the integration of specific targeting ligands, and regulate the release of antigens/adjuvants by incorporating sensitive moieties. There are three main types of polymers-based tumor nanovaccines: cationic polymers-based nanovaccines, stimuli-responsive polymers-based nanovaccines, and targeted ligand modified polymers-based nanovaccines [81, 82].

Polymeric micelles, which are nanoparticles formed by self-assembly of amphiphilic block copolymers, have been extensively studied for their potential in cancer therapy [83, 84]. These micelles have a unique core-shell structure, where the hydrophobic parts of the copolymer form the inner core, while the hydrophilic chains create an outer shell. The core of the micelles is often used to encapsulate poorly soluble compounds, while the outer shell can be modified for targeted delivery [85]. Micelles have shown great promise in cancer immunotherapy, as highlighted in comprehensive reviews [86]. For instance, in one study, linear polyethyleneimine-based nano micelles were used to encapsulate siRNA, and it was observed that dendritic cells expressing CD11c and PD-L1 efficiently took up these nano micelles in an ovarian cancer mouse model [87]. Another research work utilized cationic self-assembly micelles composed of polypeptides to load a model antigen (chicken ovalbumin, OVA), an adjuvant (poly I:C), and a siRNA (STAT3 inhibitor). These micelles were specifically targeted to immunosuppressed dendritic cells in the tumor microenvironment, resulting in the activation of dendritic cells, priming of cytotoxic T-lymphocytes, and improved survival in a melanoma mouse model [88].

#### Inorganic nanoparticles

Inorganic nanoparticles are nanostructures composed of elements and typically range in size from 1 to 100 nm. Their unique properties, such as great ease in functioning on the surface and adjusted in terms of size, shape, and charge, make them well-suited for delivering drugs. The formulation of a protein corona around the nanoparticle surface is one important discrepancy between inorganic nanoparticles and other delivery systems [89]. Researchers like Fogli and colleagues have utilized this phenomenon by exposing gold and silica nanoparticles to cancer cell lysates, resulting in the formation of a protein corona. This corona enhances the immune response by promoting the proliferation of lymphocytes mediated by dendritic cells [90]. Similarly, Zhao and co-workers employed superparamagnetic iron oxide nanoparticles as a platform for vaccine delivery and induction of immunogenicity. They observed interactions with cytokine secretion in macrophages and dendritic cells in vitro, as well as tumor growth inhibition in vivo. Co-delivering the iron oxide nanoparticles with ovalbumin led to an increased immune response and suppression of CT26 tumors. The iron oxide nanoparticles effectively stimulated immune cells and cytokine production, fostering robust immune reactions [91]. These findings highlight the promising potential of inorganic nanoparticles in the field of tumor immunotherapy. Additionally, nanomaterial-based tumor photo-thermal therapy (PTT) has gained attention as a non-invasive and site-specific treatment option. However, simply deploying PTT might not suffice to prevent tumor progression, metastasis, and recurrence. To address this, Wang and colleagues employed surface-functionalized copper sulfide nanoparticles that adsorbed tumor antigens and induced hyperthermia through thermal mediation. The modified nanoparticles, in combination with an immune checkpoint blocker and hyperthermia, resulted in increased levels of inflammatory cytokines, mobilization of CD8 + T cells within the tumor, and suppression of primary and secondary tumors in a breast cancer model [92]. In another study by Zhou et al., PEGylated MnFe2O4 nanoparticles decorated with ovalbumin and loaded with the immunoadjuvant R837 were developed for the management of breast cancer. These inorganic nanoparticles elicited substantial immune responses both in vitro and in vivo. Laser irradiation in combination with the nanoparticles led to the reduction of systemic immunosuppression, downregulation of M2-associated cytokines, inhibition of tumor growth, and prevention of lung metastases, significantly improving survival rates [93].

#### Virus-like particles (VLPs)

Throughout the years, different types of viral vectors, including lentivirus, retrovirus, adenovirus (AV), and adeno-associated virus (AAV), have been employed for delivery of functional nucleic acids into cells and tissues. Among these vectors, lentivirus and retrovirus have shown remarkable capabilities in integrating their genes into the genome, enabling long-term and stable expression of foreign genes. AAV, on the other hand, is a highly potent tool for studying gene function in vivo for its edges in safety, low immunogenicity, and sustainability for long-term gene expression [94]. By combining the AAV vector with transposon and CRISPR systems, the edit to CD8 + T can achieve nice efficiency, leading to the identification of new targets for tumor immunotherapy [95]. Additionally, AAV can be utilized to transfer CRISPR activation molecules, enhancing the expression of specific target genes in vivo and improving immune recognition and clearance [96]. However, AAV has limited capacity to carry foreign genes, while such drawback can be compensated nicely with AV [97]. AV, with its broad infection range, high efficiency, ease of use, and non-integration into the host genome, has found wide applications in gene transduction, gene therapy, vaccination, and other fields [98]. Notably, AV-based vaccines have a shorter development timeline compared to traditional inactivated and attenuated vaccines, making them particularly advantageous for fast solution for the confinement of infectious diseases, such as the COVID-19 pandemic [98].

The AV-based vaccine employs AVs as carriers, wherein the protective antigen gene is genetically incorporated into the AV genome. This allows the vaccine to exhibit antigenicity without posing any viral toxicity, thereby triggering a specific immune response. Currently, a novel AV-based vaccine called Ad5-nCoV has integrated the spike protein (S) gene of SARS-CoV-2 into the genome of replication-deficient human AVs type 5 (Ad5) for delivering effective immunity against SARS-CoV-2 infection. Typically, SARS-CoV-2 enters the body through the nasopharynx [99]. Though effectively intramuscular injection guarantees the effective activation of systemic immunity, no guarantee can be granted for complete elimination of local infections. Hence, intranasal inoculation is employed to confer immunity to cells in the nasal cavity and pharynx [100]. Feng et al. developed a codon-optimized AVs vaccine (Ad5-S-nb2) that grant one month long immune response in mice and rhesus macaques with only one single shot. Intramuscular inoculation induces systemic humoral and cellular immunity, whereas the influence imposed by intranasal inoculation to cellular immunity is generally weaker [101].

Nucleic acid vaccines offer distinct advantages in vaccine development compared to inactivated or attenuated virus vaccines. These include a simple preparation process and a shorter manufacturing time [102]. The use of viral vector-based vaccine delivery systems allows for the protection of nucleic acid antigens within the body, overcomes barriers posed by multilayer cell membranes and facilitating their delivery to antigen-presenting cells [103]. Additionally, acquirement of viral vectors requires no effort as one can easily obtain them in vitro cell culture, resulting in low production costs and high possibility in production in large scale. Furthermore, what excites us the most is that, with the support of advanced molecular biology techniques, viral vectors can be utilized as a versatile vaccine platform for the effective delivery of antigen molecules, generating robust and protective immunity against various infectious diseases. However, there are still some limitations associated with viral vectors. For instance, most viral vectors have limited targeting capabilities towards disease sites, potentially leading to adverse reactions in normal tissues and cells. Moreover, certain viral proteins can incur the immune response, which may imterfer both initial and subsequent immunizations [104].

#### MOFs

MOFs (Metal-Organic Frameworks), stands as a type of emerging nanomaterial, enjoying the edges from both inorganic and organic materials. They possess desirable characteristics such as good crystallinity, tunability, and porosity. Due to their porous nature, MOFs have been utilized in tumor immunotherapy for delivering various cargoes including small-molecule drugs, proteins, nucleic acids, and more [105]. In a specific study conducted by Zhang and colleagues, pH-responsive MOF vaccines carrying tumor-associated antigens (TAA) were developed. The MOFs exhibited degradation in the acidic environment of endosomes/lysosomes, leading to the release and escape of the TAA. Moreover, the modification of CpGs (a type of adjuvant) further enhanced the immune response of CD8 + T cells [106]. Additionally, certain types of MOFs have been explored as heat-sensitive agents for Photodynamic Therapy (PDT). Research has demonstrated that MOF-induced PDT induces larger amount of release in tumor antigens, which in turn activate the immune system. Furthermore, cationic MOFs have the ability to effectively adsorb adjuvants like CpGs, facilitating uptake by DCs and promoting antigen cross-presentation [107].

### Factors impact LNs’ targeting of Nanovaccines

The characteristics of nanovaccines, such as size, molecular weight, charge, shape, and modified ligands, play a significant role in determining their targeting and retention in lymph nodes [52]. Studies have shown that those nanovaccines which are composed by polymeric materials with size smaller than 50 nm enjoys easier transportation to lymph nodes through interstitial flow [108]. This is due to the fact that the only way larger particles can target lymph nodes is either through cell-mediated transportation or through hydrodynamic swelling effect caused by injection, while particles smaller than 20 nm are capable of returning to the peripheral blood even after entering the lymph nodes. However, the optimal size for targeting varies depending on the materials used. Ranging from 10 to 100 nm is generally believed to be ideal for lymphatic uptake [109]. Moreover, uptake capability of the lymphatic system is also determined by molecular weight of the injected compounds [110]. The surface charge of nanomaterials also plays a role in lymph node targeting, with neutral or negatively charged particles draining more freely to lymphatic vessels than positively charged ones, which are typically trapped at the injection site [83]. However, it is possible for positively-charged particles to interact with the cell membrane and be taken up by dendritic cells, which would eventually result in antigen cross-presentation and T-cell activation [84]. The shape of nanomaterials can influence how they target and accumulate in lymph nodes. According to Mueller and colleagues, a cylindrical shape (80 × 180 nm) in PRINT hydrogels platform is more efficient at draining to lymph nodes and accumulating over time (48 h) than traditional spherical nanoparticles [85].

Modifying vaccines with ligands that target specific receptors in the lymphatic system can increase their uptake and interception in the lymph nodes. Various receptors have been identified as potential targets, including Lymphatic vessel endothelial hyaluronan receptors and vascular endothelial growth factor receptors (VEGF receptor 3) expressed on lymphatic endothelial cells, peripheral lymph node address on high endothelial venules, and the mannose receptor on human lymphatic endothelium [87, 88, 111]. Serving the purpose of targeting DCs in LNs for cancer immunotherapy, the scavenger receptors class B1 has also been utilized, and one can expect its potential in a multitargeting nanovaccines for treating tumors and lymphatic disorders [112–114].

In summary, the diverse physicochemical and pharmacokinetic characteristics of various nanomaterials can be customized to target LNs and enhance immune responses. As a result, the development of functional nanovaccines with specific purposes is a promising avenue of research.

## Nanovaccines-based combination other therapy

**Fig. 4:**
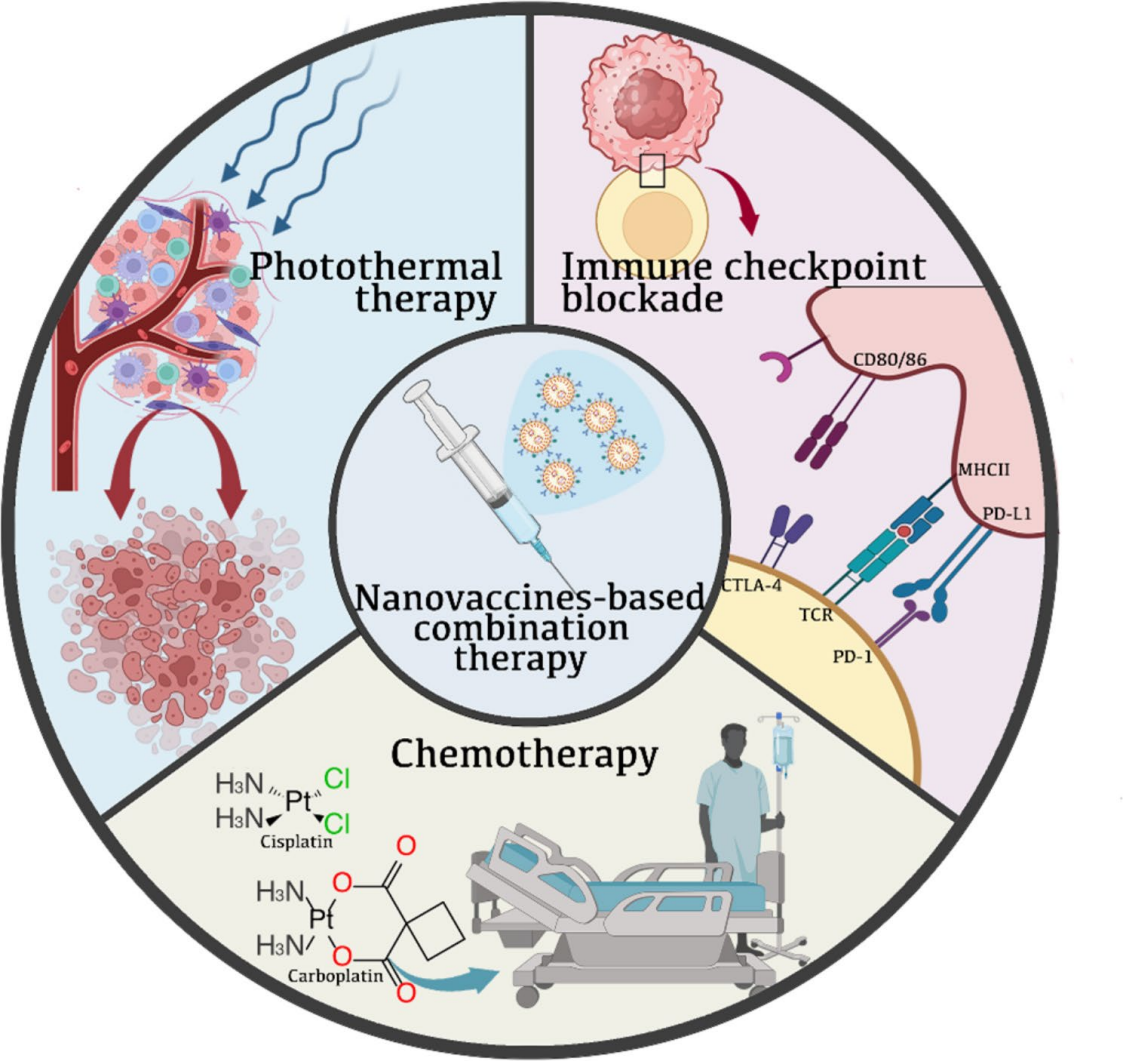
NanovaccinesNanovaccines combined with immunotherapy, chemotherapy and photothermal therapy

The effectiveness of nanovaccines used alone to treat tumors is often limited due to the diversity of tumor cells [115, 116]. Combining nanovaccines with other therapies can significantly enhance their anti-tumor effects through a synergistic mechanism. Researchers have developed a range of combination immunotherapies based on nanovaccines in recent years, among which have shown promising results in enhancing cancer therapy [116]. This article will explore the use of nanovaccines-based combination other therapies, specifically in the context of immune checkpoint blockade therapy, chemotherapy, and photothermal therapy (Fig. 4).

### Combination of nanovaccines and ICBs

The therapy of immune checkpoint blockade (ICBs), which includes PD-1, PD-L1, and CTLA-4, has achieved remarkable success in the field of tumor immunotherapy [117, 118]. However, due to the heterogeneity of tumors, one can barely expect nanovaccines alone to be omnipotent in treating various kinds of tumors. It has been demonstrated that the combination of nanovaccines and ICBs therapy might render better synergistic antitumor immunotherapy efficacy [119, 120]. Researchers have developed a variety of nanovaccines-based combination immunotherapies, including those that combine with PD-1, PD-L1, and CTLA-4 antibodies [120]. For example, researchers have used ultrasound-responsive nanovaccines and nano-discs vaccines combined with anti-PD-1 or anti-PD-L1 antibodies to significantly increase tumor-infiltrating CD8 + CTLs, natural killer (NK)1.1 cells, and CD8 + CTLs to Treg ratio, suppress tumor growth, and extend the survival time of mice bearing tumor [121]. In addition, combination of mucin 1 (MUC1) mRNA nanovaccines with anti-CTLA-4 has also demonstrated enhanced antitumor effects against triple-negative breast cancer by improving the immunosuppressive tumor microenvironment (TME) and increasing CD8 + T cells, IFN-γ, and IL-12 levels [121].

### Combination of nanovaccines and chemotherapy

Nanovaccines can be combined with chemotherapy to improve the effectiveness of cancer treatment [105, 122]. Chemotherapy is still widely used in cancer therapy and has been effective in treating advanced and metastatic cancer. The combination of nanovaccines and chemotherapy has been gaining attention in recent years. For example, Shan et al. developed a nanovaccines that utilized gene modification to integrate an OVA peptide to the surface of a natural hepatitis B core protein nanocage [105]. This nanovaccines, when combined with low dose paclitaxel, showed a stronger antitumor effect than either treatment alone. In addition, this treatment combination was successful in stopping the spread of tumors in mice with B16-OVA melanoma. Another example of combining nanovaccines with chemotherapy is the use of a modified α-Al2O3 nanovaccines called α-Al2O3-UPs with low dose epirubicin (EPB). This treatment combo demonstrated a synergistic antitumor effect of combining chemotherapy and immunotherapy [122], with the median survival time was significantly prolonged to 63 days with the combination treatment compared to 37 days in the EPB group.

### Combination of nanovaccines and PTT

Furthermore, the combination of nanovaccines and photothermal therapy (PTT) has gradually risen to be a persuasive approach for ameliorating the therapeutic outcome of cancer treatment [123]. PTT is a non-invasive method that has prevailed for treating different types of tumors, including breast cancer, lung cancer, and melanoma [124–128]. The hyperthermia generated by photosensitizers (PS) upon proper light irradiation can effectively induce tumor cell necrosis or apoptosis. It is important to note that PS has almost no cytotoxicity without laser irradiation.

Additionally, PTT has been shown to enhance infiltration of lymphocytes into tumors. Combining PTT with nanovaccines has proven to be a powerful approach to treating cancer. For example, Huang et al. developed a mesoporous silica nanoparticle-based nanovaccines that contained polydopamine (PDA) and was conjugated with thiolated OVA for PTT-immunotherapy combination therap. Upon NIR light irradiation, the PDA produced hyperthermia that not only exterminated tumor cells but also stimulated the release of antigens, leading to a strong antitumor immune response. This approach effectively eradicated primary tumors and lower the possibility of relapse for tumor after a single administration and laser irradiation. Similarly, Xiao et al. developed a nano system that integrated OVA-induced immune response, PDA-based photothermal ablation, and MnO2-driven MRI of LNs for combination therapy [129]. This vaccine not only exhibited the most powerful immune response with the highest anti-OVA IgG and the IFN-γ secretion, but also showed great potential in tracking DC migration. Such nano system provided solid proof for the synergistic antitumor effect in B16F10 melanoma mice and preventing tumor metastasis.

## Conclusion

The use of nanotechnology has opened up a promising avenue in cancer immunotherapy, particularly through the use of tumor nanovaccines that integrate nano-materials and biomedical nanotechnology. With significant progress made in this area, recent advances in tumor nanovaccines have been summarized, including various types of nanocarriers such as liposomes, polymer-based NPs, inorganic nanocarriers, VLPs, MOFs and combination immunotherapy-based nanovaccines. Among these, liposomes stand out due to their simple composition, mature industrial manufacturing technology, and potential for future clinical translation and marketization. In this article, the pre-clinical experiments of various nano-materials were described, while the clinical trials were listed in table form. Therefore, less is presented here about clinical trials. Overall, cancer immunotherapy has become an increasingly promising strategy, and the use of nanovaccines offers exciting new possibilities for the future of cancer treatment.

Although tumor nanovaccines have made remarkable progress, there are still several crucial issues that need to be think thoroughly before their potential clinical application. Firstly, the biocompatibility and biodegradability of nanocarriers, particularly inorganic nanocarriers, yet need to be further investigated. Secondly, it is challenging to identify appropriate tumor associated antigens. Thirdly, for concreteness of the experiment, the effectiveness needs to be further evaluated as we need to take into account of the dosage ratios and discrepancy between animal models and clinical cancer patients. Moreover, side effect of combination therapy needs to be fully understood before the application. Therefore, achieving the clinical translation of tumor nanovaccines still requires a considerable amount of work.

## Abbreviations

APCs: Antigen-presenting cells
CAR: Chimeric APantigen receptor
TILs: Tumor-infiltrating lymphocytes
CTLA-4: Cytotoxic T-lymphocyte-associated protein 4
PD-1: Programmed cell death protein 1
CRS: Cytokine release syndrome
DCs: Dendritic cells
CTLs: Cytotoxic T lymphocytes
iDCs: Immature DCs
MHC: Major histocompatibility complex
mDCs: Mature DCs
TCRs: T-cell receptors
IFN-𝛾: Interferon-𝛾
SLN: Sentinel lymph node
LNM: Lymph node metastasis
PTT: Photo-thermal therapy
VLPs: Virus-like particles
AAV: Adeno-associated virus
AV: Adenovirus
TAA: Tumor-associated antigens
PDT: Photodynamic Therapy
ICBs: Immune checkpoint blockade
NK: Natural killer
PTT: Photothermal therapy
PDA: Polydopamine
